# Bioactive Compounds and Antioxidant Capacity of *Rosa rugosa* Depending on Degree of Ripeness

**DOI:** 10.3390/antiox7100134

**Published:** 2018-10-03

**Authors:** Ahlam Al-Yafeai, Peter Bellstedt, Volker Böhm

**Affiliations:** 1 Institute of Nutritional Sciences, Friedrich Schiller University Jena, Dornburger Straße 25-29, 07743 Jena, Germany; Ahlam.Al-Yafeai@uni-jena.de; 2 Department of Biology, Science Faculty, Ibb University, Ibb, Yemen; 3 Institute of Organic Chemistry and Macromolecular Chemistry, Friedrich Schiller University Jena, Humboldtstraße 10, 07743 Jena, Germany; peter.bellstedt@uni-jena.de

**Keywords:** gazaniaxanthin, NMR (nuclear magnetic resonance), L-TEAC

## Abstract

Maturity stage affects the bioactive compounds as well as the antioxidant capacity in the fruit. This study was designed to identify and quantify carotenoids, as well as to evaluate vitamin E, vitamin C, antioxidant capacity and total phenolic compounds of *Rosa rugosa* hips at different degrees of ripeness. HPLC (high performance liquid chromatography) analysis showed different types of carotenoids at different stages of maturity of *R. rugosa* hips with significant differences (*p* ˂ 0.05), where the maximum concentration was observed at late harvesting. In the hips investigated, only α-tocopherol was detected, the maximum concentration of both vitamin E and vitamin C was obtained in the orange hips with significant difference (*p* ˂ 0.05). On the other hand, the highest hydrophilic and lipophilic TEAC (Trolox equivalent antioxidant capacity) values, as well as total phenolic contents, were determined in the mature hips (red colour) with significant difference (*p* < 0.0001) and (*p* < 0.001) respectively, whereas ORAC (oxygen radical absorbance capacity) showed lower activity in the mature hips with significant difference (*p* ˂ 0.05). Late harvesting is recommended if a high content of carotenoids is desired, while harvesting should be carried out earlier if a higher vitamin E and vitamin C content is desired, which in turn affects the antioxidants capacity.

## 1. Introduction

Recent developments in the fields of health and food have led to a renewed interest in natural compounds with antioxidant potential. A diet rich in antioxidant components has potential effects on the human health by reducing the risk of various diseases, for example cardiovascular diseases, cancers and age-related macular degeneration [1]. The selection of species/varieties with high contents of bioactive compounds and harvesting at the optimum time can promote the increase in the uptake of bioactive compounds from the fruits and vegetables. The genus Rosa contains over 100 species that are widely distributed mostly in Europe, Asia, the Middle East and North America [2]. *R. rugosa* hips resemble tomatoes with a 2–3 cm diameter and often a shorter height than their diameter. During maturation, the hips’ colour begins to change on their sunny side and become completely coloured over time [3,4]. In the same vein, rosehips have a fairly long maturation period, harvesting once a year in the autumn after the first night frost. In recent decades, the rosehip fruit has been increasingly studied for its preventive properties [5]. Previous studies reported that rosehips contain higher amounts of various bioactive compounds than several other fruits such as: carotenoids, vitamins (particularly vitamins C, E and provitamin A), flavonoids, tannins and fatty acids [3,6,7]. Moreover, Al-Yafeai et al. (2018) [3] have reported that rosehips extract contained four (*Z*)-lycopene isomers. Furthermore, rosehips extract was able to scavenge reactive oxygen species (ROS) [8]. The *Rosa multiflora* hips have been traditionally utilised as herbal remedies and nutritional supplements for diseases treatment, including osteoarthritis, rheumatoid arthritis, chronic pain as well as in the cold and inflammation diseases [9]. Although *Rosa rugosa* is known to produce the most abundant and best tasting hips, most food products are based on the hips of *R. canina*, although they are small compared with the *R. rugosa* hips [10]. In addition, *Rosa canina* hips have been largely utilised in traditional folk medicine. Although the carotenoids composition as well as vitamin E contents in mature *R. rugosa* have been previously described [3], there is no work presenting how ripening stages influence *R. rugosa* hips composition as well as antioxidant capacity (AOC). This study aimed to address the following research question: effect of ripening times on the carotenoid, vitamin E and vitamin C contents as well as on antioxidant capacity in *R. rugosa* hips and how to improve levels of these bioactive compounds by choice of harvesting time. On the other hand, the present study was also designed to identify and quantify the type of (*Z*)-isomers of lycopene.

## 2. Materials and Methods

### 2.1. Chemicals

All chemicals and buffer salts were of analytical quality and the solvents for chromatography were of HPLC grade. HPLC grade water was produced using a MicroPure instrument (Thermo Electron LED GmbH, Niederelbert, Germany). Moreover, the chemicals were of the highest quality available (95–99%) and were used without purification. The standards of carotenoids (97–99%) were from CaroteNature (Münsingen, Switzerland), (*all-E*)-rubixanthin was supplied by DSM, Kaiseraugst, Switzerland. Pure tocotrienols (>97%) and tocopherols (>95%) were purchased from Davos Life Sciences (Singapore, Singapore) and Calbiochem (Darmstadt, Germany), respectively. dl-α-tocopheryl acetate (>96%), 2,2’-azinobis-(3-ethylbenzothiazoline-6-sulphonic acid) diammonium salt (ABTS), 2,2’-azobis-(2-amidinopropane) hydrochloride (AAPH), phosphate buffered saline (PBS; pH 7.4, 75 mM), 6-hydroxy-2,5,7,8-tetramethylchroman-2-carboxylic acid (Trolox), HCl and Folin-Ciocalteu phenol reagent (FCR) were obtained from Sigma-Aldrich (Taufkirchen, Germany). Thiourea, meta-phosphoric acid and copper (II) sulphate (CuSO4) were purchased from Merck (Darmstadt, Germany). L (‏+)-ascorbic acid (AA), fluorescein and 3, 4, 5-trihydroxybenzoic acid (gallic acid) were purchased from Fluka (Buchs, Switzerland). Na_2_CO_3_, NaCl, NaOH, CH_3_COONa∙H_2_O, Na_2_HPO_4_∙2H_2_O, NaH_2_PO_4_∙2H_2_O and Trichloroacetic acid (TCA) were obtained from VWR (Darmstadt, Germany).

### 2.2. Samples Description

One kg of *Rosa rugosa* Thunb. hips were harvested at Dorndorf-Steudnitz 51°00’ N, 11°68′ E at different ripening stages from different bushes of *Rosa rugosa* in late summer to early autumn of 2016. The average temperature was between 26 °C and 17 °C and the hips were picked by hand once and then separated into three groups depending on the degree of ripeness (green, orange and red). For analysis, the hips were cleaned, first rinsed thoroughly for 1–2 min with tap water, then 1 min with distilled water. Before homogenization, the seeds, calyx and stem were removed, then samples were ground for 20 s with a mill (Retsch Grindomix GM 200, Haan, Germany) and stored at −25 °C until analysis.

### 2.3. Carotenoids and Vitamin E Determinations

#### 2.3.1. Extraction Procedure

Extraction of carotenoids and vitamin E from raw *R. rugosa* hips at different ripening times was performed by methods previously described by Al-Yafeai et al. (2018) [3]. 35 mL of MeOH/THF (1/1, *v/v*), 200 mg magnesium oxide and 200 mg sodium sulphate were added to 500 mg of *R. rugosa* hips. The homogenization was carried out for 5 min by using ultra turrax, the supernatant was filtered and collected while the residue was extracted again, at least two times. The collected supernatants were concentrated in a rotary evaporator and the final volume was 5 mL. Saponification is a necessary step to determine xanthophyll contents in rosehips, one advantage of the saponification is that it avoids the problem of unwanted lipids and other interfering substances and to hydrolyse carotenoid esters. Saponification was conducted by using 10% methanolic KOH for 60 min at room temperature. All procedures were carried out under dim light to sidestep the photo-degradation. HPLC analysis of carotenoids, lycopene isomers and vitamin E was realised immediately after extraction.

#### 2.3.2. Carotenoids Identification and Quantification

HPLC analysis of carotenoids was performed by using reversed-phase HPLC with a photo diode array (PDA) detector according to the method previously described by Al-Yafeai et al. (2018) [3]. The method was reproducible and specific, the identifications were achieved by comparison with external standards, or compounds were tentatively identified by comparison of retention times and DAD absorption spectra and mass spectra. On the other hand, the quantifications were done using a 5-point calibration curve (r^2^ ≥ 0.999) of external standards. The limits of detection (LOD) and limits of quantification (LOQ) of the carotenoids were calculated using the baseline noise signals: LOD was supposed to a signal-to-noise (S/N) ratio of 3:1, LOQ to that one of 10:1.

#### 2.3.3. Analysis of Lycopene Composition

To increase the reliability of measures, chromatographic separation of the lycopene isomers was achieved using the isocratic C30-HPLC method according to the method reported by Al-Yafeai et al. (2018) [3]. The identification was carried out by comparing their retention times with those of external standards, while the quantification was achieved by 3-point calibration curve (r^2^ ≥ 0.999) of external standards of *(all-E)-*lycopene, *(13Z)-*lycopene, *(9Z)-*lycopene and *(5Z)-*lycopene by using specific extinction coefficients [11].

#### 2.3.4. Isomerisation and NMR Analysis

As mentioned previously, (*5′Z*)-rubixanthin (gazaniaxanthin) is the main *(Z)-*isomer of *(all-E)-*rubixanthin as well as is one of the important features that characterised *R. rugosa* compared to *R. canina*. The major problem in isolating *(5’Z)-*rubixanthin using the fractionation method [3] is that the concentration was too low. In addition, invisible compounds having a molecular weight of more than 1000 *m/z* were simultaneously eluted. An alternative method for confirming our results was using isomerisation of *(all-E)-*rubixanthin and checking the isomers by using NMR according to a method reported by Arpin & Liannen-Jensen (1969) [12] and Kishimoto et al. (2005) [13]. Approximately 1 mg (*all-E*)-rubixanthin was dissolved in CDCl_3_ and immediately subjected to NMR analysis. Special attention was made to circumvent exposure to sun-/daylight. All NMR experiments were performed on a 600 MHz Bruker Avance III spectrometer equipped with a 5 mm triple resonance cryogenic probe. Sample temperature was set to 293 K. The referencing was carried out using the residual 1H solvent peak of CDCl_3_. The assignment was based on literature data [13] as well as predicted spectra (ACD Labs NMR Workbook 2017.2.1) and was confirmed by standard NMR experiments (^1^H; ^1^H,^13^C-HSQC-DEPT (heteronuclear single-quantum correlation-distortionless enhancement by polarization transfer); ^1^H, ^1^H-COSY (correlation spectroscopy), ^1^H, ^1^H-TOCSY (total correlation spectroscopy)). Isomerization of *(all-E)-*rubixanthin was induced by exposition to sun-/daylight for 10 min. In addition to the aforementioned 2D experiments, selective 1D COSY, TOCSY and NOESY spectra were collected to assign the newly-formed signals in the aliphatic region. 3D lowest energy conformers were generated with Marv in Sketch (Version 17.1.2.0; ChemAxon Ltd., Budapest, Hungary) employing the MMFF94 force field [14].

#### 2.3.5. Vitamin E Identification and Quantification

The chromatographic separation of the tocols was achieved using a normal-phase HPLC equipped with a fluorescence detector according to the method reported by Al-Yafeai et al. (2018) [3]. The identification of individual tocochromanols was achieved by comparing their retention times with those of external standards and tocols were quantified by 6-point calibration curve (r^2^ ≥ 0.999) of external standards. The total contents of vitamin E were estimated as the sum of all individual tocopherols and tocotrienols determined by HPLC analysis.

### 2.4. Vitamin C

#### 2.4.1. Extraction Procedure 

For vitamin C analysis, 0.5 ± 0.05 g of *R. rugosa* hips were extracted two times by using meta-phosphoric acid (4.5%). Following this, the samples were shaken for 1 min by vortex and centrifuged for 5 min at 3000× *g*.

#### 2.4.2. Quantification of Vitamin C

Vitamin C contents were determined based on reaction of 2,4-dinitrophenylhydrazine (DNP) with ketone group of dehydroascorbic acid under acidic conditions to form red osazone derivatives, which were subsequently measured at 540 nm. The experiment was performed in triplicate according to Al-Duais et al. (2009) [15]. The standard solutions of ascorbic acid were prepared in the range of 0.5–50 µg/mL. A standard curve of ascorbic acid reference was constructed and the ascorbic acid contents of *R. rugosa* hips were determined using the equation of the linear regression and are expressed as mg/100 g.

### 2.5. Antioxidants Capacity and Total Phenolic Contents

#### 2.5.1. Extraction Procedures

Samples were prepared in triplicates, approximately 1 ± 0.05 g of *R. rugosa* hips were weighed into 15 mL centrifuge tubes. For hydrolysis, 1 mL hydrochloric acid (1 M) and glass beads were added to the hips. Afterwards, 1 mL sodium hydroxide (2 M in 75% MeOH) and 1 mL meta-phosphoric acid (0.75 M) were added. All steps were accompanied by shaking for 30 s and heating in a water bath (30 min 37 °C). Following the centrifugation (5 min, 3000× *g*), the supernatant was collected in a 25 mL volumetric flask, while the residue was extracted again with 5 mL of 70% methanol, this step was accompanied by shaking for 1 min and centrifugation (3000× *g* for 5 min). Finally, the extraction was repeated twice and for the final volume, the volumetric flask was filled up with 70% methanol. For the lipophilic test, analysis was performed with carotenoid extracts that have been mentioned above. The collected supernatants were concentrated in a rotary evaporator and the final volume was 5 mL. The resulting solutions were re-dried under a stream of nitrogen gas and re-dissolved in hexane. An aliquot of 1.5 mL was centrifuged again (20000× *g*, 5 min) and then different dilutions were prepared.

#### 2.5.2. Determination of Total Phenolic Contents (TP)

Total phenolic contents, including other polar antioxidants (e.g., vitamin C), were assessed using the Folin-Ciocalteu method [16]. This method is based on a colorimetric oxidation/reduction reaction. Using a 96-well microtiter plate, 30 μL of HPLC water (blank), gallic acid monohydrate standard (8.51–170.12 mg/L) and methanol/water extract (70/30, *v/v*) were added. Following this, 150 μL of Folin-Ciocalteu’s 1:10 diluted reagent and 120 μL of sodium carbonate solution (75 g/L) were gradually mixed with the samples. In darkness for 2 h and at ambient temperature, samples were incubated, the absorbance measurement was performed at 740 nm in the microplate reader at 30 °C. The TP is expressed as gallic acid equivalents (GAE) in mg/100 g.

#### 2.5.3. Hydrophilic Trolox Equivalent Antioxidant Capacity (H-TEAC) Assay

The hydrophilic TEAC assays use intensely coloured cation radicals of ABTS^•‏+^ to test the ability of antioxidants to quench radicals and this was performed according to the method previously described [16]. The ABTS^•‏+^ radical was prepared using 10 mL ABTS solution (7 mM) and 10 mL of potassium peroxodisulfate solution (2.45 mM). In the darkness and at ambient temperature, the stock solution was incubated for 24 h, ABTS^•‏+^ working solution was freshly diluted with phosphate buffer (75 mM, pH 7.4). For analysis, in the 96-well microtiter plate, 20 μL of HPLC water (blank), Trolox standard (12.5–250 μM)) and methanol/water extract (70/30, *v/v*) were mixed with 200 mL ABTS^•‏+^ working solution. The drop of absorption of the stable radical ABTS^•‏+^ during the reaction with antioxidants is measured at 734 nm, the antioxidant capacity is expressed as Trolox equivalents (TE) in mmol/100 g.

#### 2.5.4. Hydrophilic Oxygen Radical Absorbance Capacity (H-ORAC) Assay

In the H-ORAC assay, the reaction between the peroxyl radical and a fluorescent probe was achieved, generating a non-fluorescent product that can be readily quantified using fluorescence. For analysis, dilution 1:100 of the stock solution of fluorescein (0.12 mM) and phosphate buffer (75 mM, pH 7.4) was utilised to prepare a working solution of fluorescein (1.2 μM). The reaction was performed by adding 10 μL of methanol/water (70/30, *v/v*) extract, 25 μL working solution of fluorescein and 100 μL buffer into the 96-well plate wells. Following this, the microplate was incubated (10 min at 37 °C), after adding 150 μL of a freshly prepared AAPH solution (129 mM), the intensity of fluorescence was measured at 37 °C for 120 min every 60 s [16]. The antioxidant capacity was evaluated by reducing the rate of the non-fluorescent product formed over time. The protective effects of the antioxidants were calculated using the integrated area under the fluorescence decay curves (AUC). The Trolox equivalents (TE) are expressed as mmol TE/100 g.

2.5.5. α-Tocopherol Equivalent Antioxidants Capacity (TEAC) Assay

The α-TEAC method, previously described by Müller et al. 2010 [17], was used to evaluate the lipophilic antioxidant capacity in *R. rugosa* hips at different ripening times. Carotenoids extracts were prepared as described above and the final volume was 5 mL. Under a stream of nitrogen gas, the resulting solutions were dried and then re-dissolved in hexane. Meanwhile, ABTS^•‏+^ radical was produced by giving ABTS solution through a filter paper coated with manganese dioxide. The working solution of ABTS^•‏+^ was freshly prepared by diluting it with phosphate buffer (pH 7.4) and the absorbance measurement was carried out (0.70 ± 0.05) at 734 nm. The reaction was performed by adding 100 µL of lipophilic extract and 1000 μL ABTS^•+^ solution into the 2 mL cuvette, followed by mixing for 30 s. Phase separation was achieved through centrifugation (200× *g*, 30 s) and the absorbance was recorded exactly after 2 min. The α-tocopherol standard solutions were used to construct a linear regression line. The working standards of α-tocopherol (3.3–81 µmol/L) were prepared daily, linearity was given over the entire 5-point standard curve (r^2^ = 0.999). The α-TEAC in the samples is expressed as mmol α-TE (tocopherol equivalents)/100 g.

### 2.6. Statistical Analysis

The data analysis was achieved using Prism program for windows, version 7.0 (Graph pad software, Inc, San Diego, CA, USA). All analyses were done in triplicate and the results are expressed as mean ± standard deviation (SD). A difference was considered statistically significant at *p* < 0.05. A one-way ANOVA (analysis of variance) followed by Student-Newman-Keuls post-hoc test (S-N-K) was utilised to compare data obtained from different analyses. Correlations were estimated using Pearson’s correlation coefficient (R) and coefficient of determination (r^2^) was used to determine the precision of methods used.

## 3. Results and Discussion

### 3.1. Carotenoids Identification and Quantification

Since most of the hips are harvested from wild plant groups, it is difficult to maintain quality aspects such as the contents of vitamins and antioxidants in raw materials during manufacturing processes. Although rosehips had more recently attracted attention because of their potential health benefits, there was little information about the changes of antioxidants properties that occur during the maturity especially in *R. rugosa*. Understanding physicochemical properties of fruits during the ripening is important to promote the levels of bioactive compounds through the selection of cultivar/species and harvest time. Due to the absence of esterified xanthophyll standards, the saponified extracts were utilised to identify and quantify carotenoids, although the damage of carotenoids cannot fully be avoided during saponification. The carotenoids characterisation and identification in the *R. rugosa* hips at different ripening degrees was achieved depending on their chemical properties and chromatographic and spectroscopic characteristics (UV-vis and MS). In addition, the mass spectrum and the abundance of molecular ion [M+H] + *m/z* results confirmed the previous findings [3].

**Figure 1:**
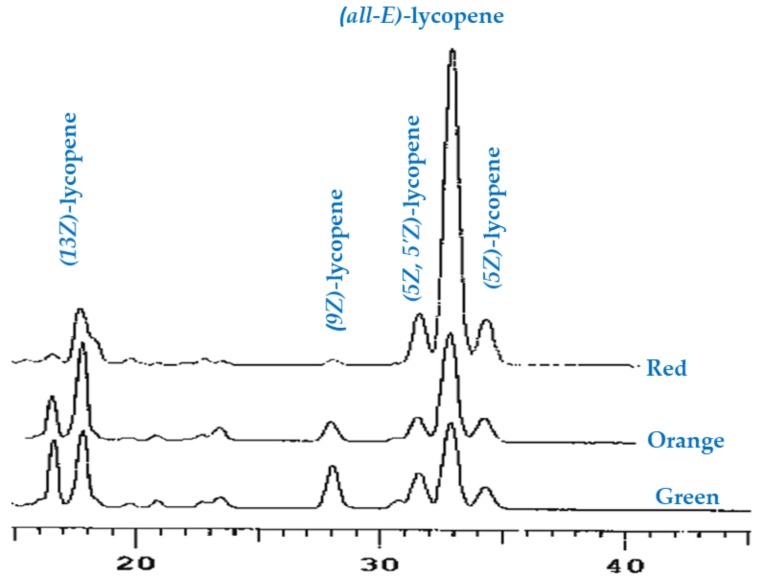
HPLC chromatograms of lycopene isomers in saponified extracts rosehips of *R. rugosa* at different ripening degrees, using a C30 column and an isocratic mobile phase.

The quantitative estimate (Table 1) showed variations between the concentrations of carotenoids in *R. rugosa* hips at different ripening degrees with significant difference (*p* < 0.05), the present study confirms our previous findings [3] in mature *R. rugosa* hips. Total carotenoids were analysed, during ripening the concentration of carotenoids increased due to the accumulations of *(all-E)-*β-carotene, *(all-E)-*rubixanthin and *(all-E)-*lycopene (Table 1). The findings of the current study are consistent with Fraser et al. (1994) [18] who suggested the concentration of carotenoids increased in tomato during ripening between 10- and 14-fold due mainly to the accumulation of lycopene (Figure 1). The total carotenoid contents were lowest in the immature stage and showed a pattern of continually increasing accumulation until the final stage of maturity, as was observed for lycopene. This is likely a result of the higher contribution of lycopene to total carotenoids, lycopene is a major carotenoid in mature rosehips [3,7]. On the other hand, Staffan et al. (2008) [19] reported that the total carotenoids content increased more than 10-fold during the eight weeks of maturation of *R. spinosissima*, while in other species of rosehips the carotenoids content increased 1.3–2.6 times during the five-week study period. Interestingly, in *R. rugosa* hips a significant increase in the total carotenoid contents (1.0-fold) was observed in orange colour, whereas a 1.5-fold increase was observed within mature stage. In the mature fruits, the carotenogenesis is controlled by particular mechanisms that differ from those in the photosynthetic tissues, therefore carotenoids are a fundamental part of the pigment-protein complexes in thylakoid [20]. This phenomenon has been attributed to an upregulation of carotenoid gene expression (phytoene synthase) with maturation. The enzyme catalyses the first committed step of carotenoid synthesis, the conversion of geranylgeranyl pyrophosphate to phytoene; phytoene serves as a precursor of lycopene from which several other carotenoid compounds are synthesized [21].

In accordance with these explanations, the present study has shown a significant decrease in the phytoene contents during the maturity (Table 1). Even though, xanthophylls such as violaxanthin, lutein, zeaxanthin and rubixanthin and carotenes such as β-carotene and lycopene were found at all stages of maturation, concentrations tended to change at different ripening stages. Likewise, (*all-E*)-β-carotene was intensively increased in the orange-red stages of maturity. In accordance with the present results, previous studies have demonstrated that higher *(all-E)-*β-carotene contents were also previously observed by Kotikova et al. (2011) [22] in the red stage in all commercial cultivars of tomatoes. *(all-E)-*β-Carotene played an important role as a photoprotective antioxidant during the photosynthesis procedure in immature fruits [23], as well as contributes to the colour of mature fruits together with lycopene [22]. Our study provides additional support for *(all-E)*- and *(Z)-*lycopene identification and quantification n rugosa hips at different ripening times. The HPLC chromatogram showed that *(all-E)-* and *(Z)*-lycopene isomers eluted at different retention times as shown in Figure 1, the peaks were determined as (*13Z)*-, *(9Z)*-, *(all-E)*- and *(5Z)*-lycopene.

**Figure 2:**
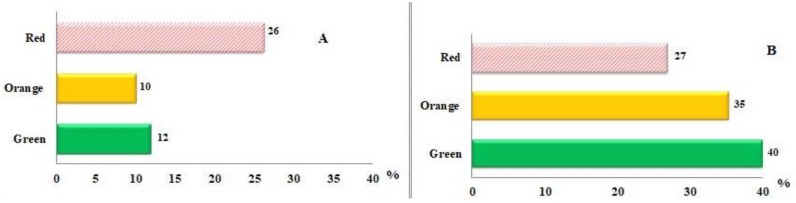
(**A**) The Percent (%) of *(all-E)-*lycopene based on total carotenoids in *R. rugosa* hips at different ripening degrees. (**B**) The Percent (%) of *(Z)-*lycopene based on total carotenoids in *R. rugosa* hips at different ripening degrees.

These results support our findings [3] and are consistent with those of other studies indicating that *R. rugosa* hips contained three types of *(Z)-*lycopene [24]. The quantitative determination of *(Z)*-lycopene isomers is presented in Table 1, the statistical analysis revealed significant differences (*p* < 0.05). *(all-E)-*Lycopene contents showed an increasing pattern of accumulation based on total carotenoids during the ripening (Figure 2A). 

**Figure 3:**
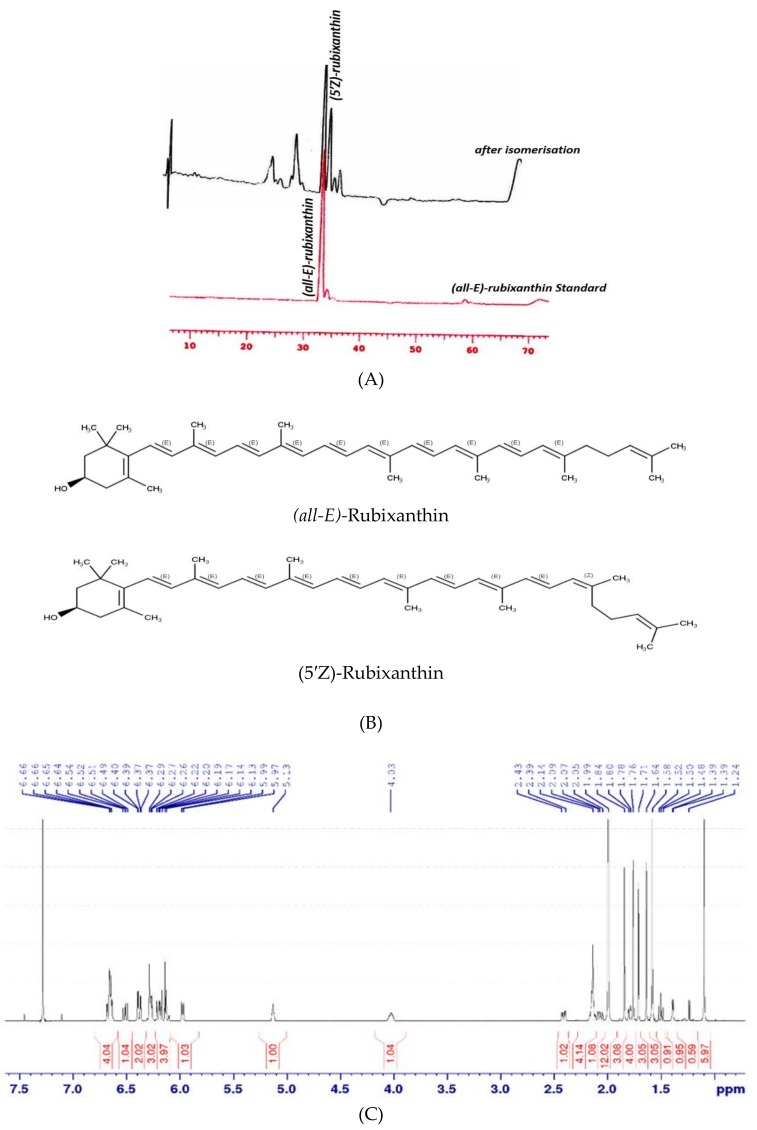

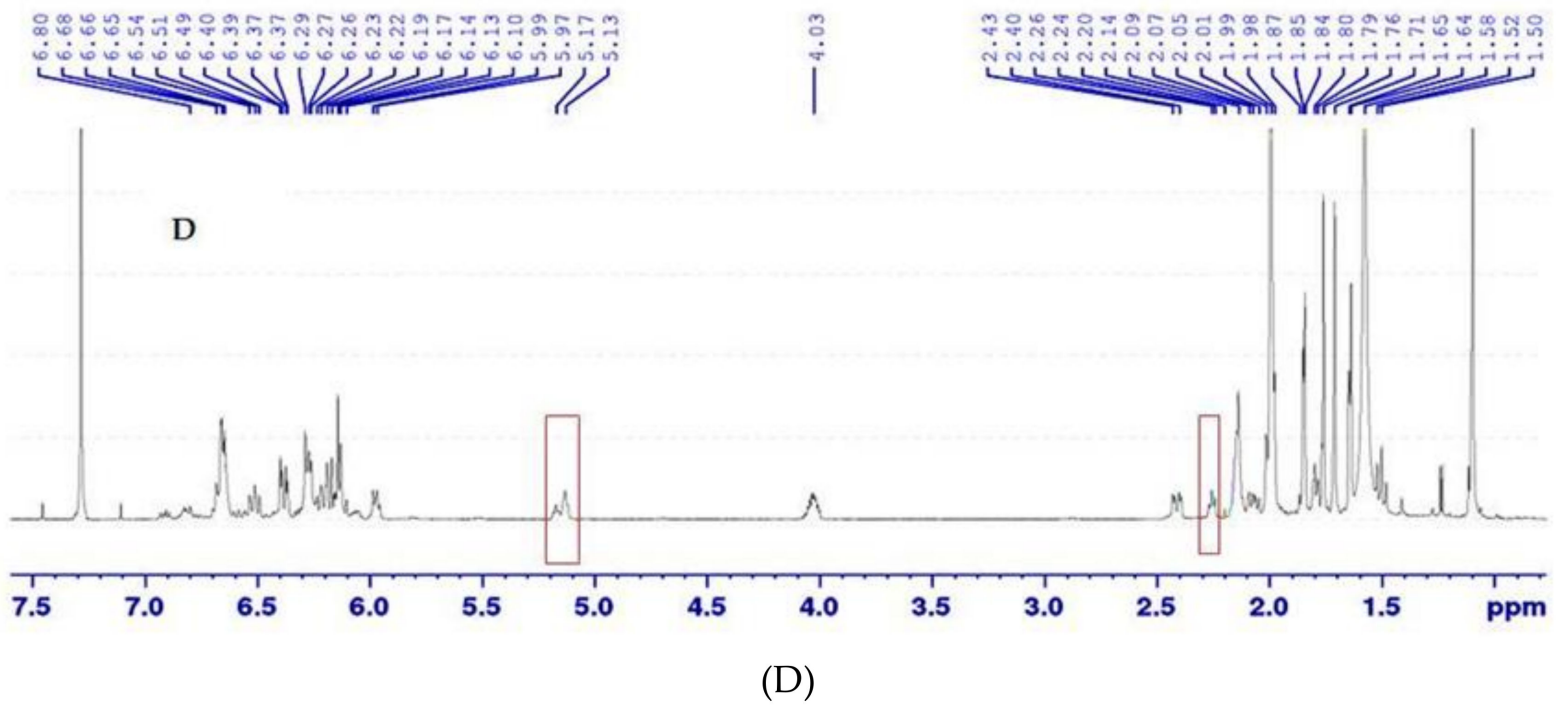
(**A**) Chromatographic separation of *(all-E)-*rubixanthin and (*5′Z)-*rubixanthin, using a C30 reversed phase column (see text for chromatographic conditions). (**B**) Structures of *(all-E)-*rubixanthin and *(5′Z)-*rubixanthin. (**C**) ^1^H nuclear magnetic resonance (NMR) spectrum (600 MHz, 293 K, CDCl_3_) of *(all-E)-*rubixanthin prior to light exposition. (**D**) ^1^H NMR spectrum (600 MHz, 293 K, CDCl_3_) of *(5′Z*)-rubixanthin after light exposition.

A possible explanation for this might be the transition of chloroplasts into chromoplasts. In contrast, *(Z)-*lycopene showed the opposite trend of accumulation compared to *(all-E)-*lycopene, such that its concentration decreased based on total carotenoids in fruits across the ripening stages (Figure 2B). In particular, the isolation of gazaniaxanthin from *R. rugosa* hips was problematic because of its low concentration. In addition, invisible compounds having a molecular weight of more than 1000 *m/z* were simultaneously eluted. For these reasons, the isomerization of *(all-E)* rubixanthin was used to confirm our results. The HPLC results obtained from the isomerization of *(all-E)-*rubixanthin by using CDCl_3_ and sun light are set out in Figure 3A. Both pigments, *(all-E)-*rubixanthin and gazaniaxanthin, showed the spectra in HPLC mobile phase with maximum absorbance at 463.3 nm and 463.3 nm, with retention times of 33.17 and 34.35 min, respectively due to only slight differences in polarity. The UV spectrum was previously described and is characterized also by the appearance of a new maximum around 330–350 nm (cis-peak), ultraviolet spectrum is moved 5-10 nm towards the shorter wavelengths, and this finding supports our previous research [3] and is also comparable to recent results [25,26].

### 3.2. Isomerisation and NMR Analysis of (all-E)- and (5′Z)-Rubixanthin

*(5′Z)-*Rubixanthin (gazaniaxanthin) has been previously described as the main isomer for (*all-E)-*rubixanthin (Figure 3B) as well as being one of the important features that characterize *R. rugosa* compared to *R. canina* [3]. For further specific identification of (5’Z)-rubixanthin immediately after isomerization, the sample was checked by NMR technique. The ^1^H spectra of (*all-E*)-rubixanthin before and after exposition to sunlight are presented in Figure 3C,D, basically two new signals appeared in the aliphatic region. Since the multiplet signal at 5.13 ppm (Figure 3C) belongs to the terminal CH proton of (*all-E*)-rubixanthin, we speculated that the new signal at 5.17 ppm (Figure 3D) was indicative of the rearrangement of the 5′ double bond from E to Z configuration. Based on selective COSY and TOCSY spectra that probe for neighbouring protons inside the same spin system, the second new signal of the light-induced isomer could be assigned to the CH_2_ group terminal to the 5′ double bond (data not shown).

**Figure 4:**
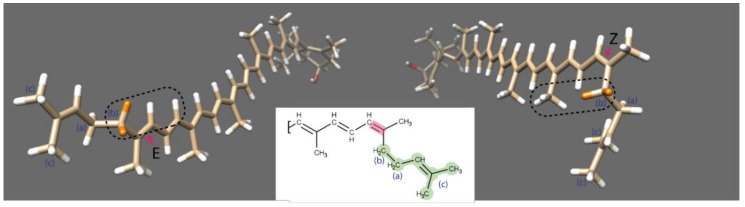
3D structures of *(all-E)*-rubixanthin (left) and *(5’Z)*-rubixanthin (right), the 5’ double bond is highlighted in pink. The protons of CH_2_ group adjacent to the 5’ double bond are marked in orange. As indicated by the dotted fields, in the *(all-E)*-rubixanthin this CH_2_ group is exposed to CH protons only, whereas in the case of the *(5’Z)*-isomer it is additionally exposed to a methyl group.

In the (*all-E*)-rubixanthin, both CH_2_ groups between the 5′ double bond and the CH group have similar chemical/electronic environments resulting in a common signal at 2.14 ppm with an integral of 4 (Figure 3C). Upon light-induced isomerisation, the signal of the CH_2_ group adjacent to the 5′ double bond slightly shifted downfield to 2.24 ppm. This shift can be attributed to the emerged spatial proximity of a CH_3_ group upon isomerisation of rubixanthin (Figure 4). Taken together, based on the NMR data we reason that exposure of (*all-E*)-rubixanthin to sunlight results in an isomerisation of the very last double bond of the conjugated double bond system in the aliphatic chain of rubixanthin forming *(5′Z)-*rubixanthin (Figure 3B). The findings of the current study are consistent with those of [12,27] who suggested that gazaniaxanthin is the corresponding 5′-cis isomer of (*all-E*)-rubixanthin.

### 3.3. Vitamin E Identification and Quantification

**Figure 5:**
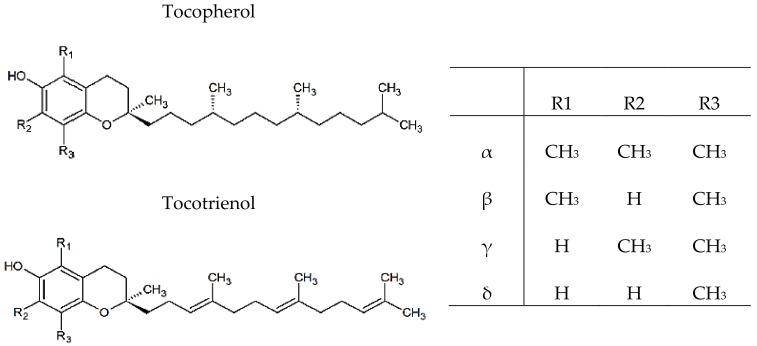
Chemical structures of tocopherols and tocotrienols.

Vitamin E is an important lipid-soluble antioxidant found naturally in eight different forms, including α-, β-, γ-, δ-tocopherol and -tocotrienol. The basic structure of vitamin E is comprising a hydrophobic polyprenyl side chain with a polar chromanol ring, tocochromanols with a fully saturated side chain are called tocopherols and those with double bonds at positions 3′, 7′ and 11′ of the side chain are tocotrienols. The specific tocopherols and tocotrienols differ by number and positions of the methyl groups in the 6-chromanol ring, resulting in the α-, β-, γ- and δ-isomers (Figure 5) [28].

α-Tocopherol is commonly referred to as vitamin E, because it is the most biologically active and widely distributed form in nature. Limited information is available concerning the contents of tocopherols and tocotrienols in rose hips especially in *R. rugosa*. To our knowledge, variations during the ripening times have not previously been investigated. In the present study, α-tocopherol was the main isomer of tocopherol in *R. rugosa* hips, this finding supports our previous research [3]. The content of α-tocopherol is likely to vary during the ripening period, the maximum concentration was achieved in the orange hips with significant difference (*p* ˂ 0.05) (Table 2).

The present findings seem to be consistent with Andersson et al. (2012) [29] who found the amounts of total tocopherols and vitamin E activity being decreased in rose hips during ripening, the change was relatively small and limited. Since α-tocopherol is the strongest tocopherol in quenching of singlet oxygen, the high concentrations of α-tocopherol participate in the stability of berries in the final stages of development [30].

### 3.4. Quantification of Vitamin C

Vitamin C (Ascorbic acid, AA) is the most powerful water-soluble antioxidant in human blood plasma, acts as a regenerator for vitamin E in lipid systems. AA is an odourless, white solid having the chemical formula C_6_H_8_O_6_ [31]. An enhanced fruit AA pool has also been suggested to be associated with improved postharvest fruit quality in hard fruit species, such as pear and apple [32,33]. Generally, rosehips are considered the most abundant natural source of vitamin C, the contents ranged between 200 and 2800 mg/100 g [34]. Thus, the medicinal value of rosehips depends largely on the vitamin C contents. The results obtained from the preliminary analysis of AA in *R. rugosa* hips at different ripening degrees are shown in Table 3, the contents ranged between 798 mg/100 g and 1090 mg/100 g. It is encouraging to compare these findings with those found by Ercisli (2007) [35] who found that contents of AA in the fresh fruits of rose species were between 706 mg/100 g and 974 mg/100 g. According to the results, the amounts of AA varied greatly at three maturity stages of *R. rugosa* hips with a significant difference (*p* < 0.05). The higher contents of vitamin C were achieved at the half-ripe (orange colour) stage with 14% of increase, whereas these contents decreased in the fully-ripe (red colour) stage by 16%. The present findings seem to be consistent with Zhang et al. (2006) [36] who found the AA contents increased at the beginning and middle of fruit growth and decreased after the development of fruits colour. Rousi & Aulin (1977) [37] reported a decreasing trend in vitamin C contents, accompanied by a steady increase in the fresh weight of the berries. On the other hand, the low vitamin C content in the plants may be due to the level of oxygen in the environment, the amount of light that reaches the plants and variations in endogenous plant growth regulators and temperature [38].

### 3.5. Antioxidant Capacity

The measurement of the antioxidant capacity of food products is a matter of growing interest because it may provide a variety of information, such as resistance to oxidation, quantitative contribution of antioxidant substances or the antioxidant capacity that they may present inside the organism when ingested [39]. Depending on the reaction mechanism the antioxidants capacity methods can be classified into two groups: hydrogen atom transfer (HAT) and electron transfer (ET). The majority of HAT-based assays measure the capability of an antioxidant to quench peroxyl radicals through transferred hydrogen atom (H) of a phenol (Ar-OH), which include the oxygen radical absorbance capacity (ORAC) assay. Whereas, ET-based assays measure the capacity of an antioxidant in the reduction of an oxidant, which changes colour when reduced. The degree of colour change is correlated with the sample’s antioxidant concentrations, (ABTS)/hydrophilic Trolox-equivalent antioxidant capacity (H-TEAC) is one of the decolourisation assays [40].

#### 3.5.1. Total Phenolic Contents (TP)

The antioxidant capacity of phenolic compounds is mainly due to their redox properties, which allow them to act as reducing agents, hydrogen donors, singlet oxygen quenchers or metal chelators [41]. Several previous studies have confirmed the presence of phenolic compounds in rosehips, Montazeri et al. (2011) [42] found that the levels of TP in *R. canina* extracts were 424.6 ± 1.8 mg GAE/g. In contrast, Denev et al. (2013) [43] reported that TP in the fruits of *R. canina* was 1934 ± 4 GAE/100 g. The maturity stage affects the TP in the *R. rugosa* hips, the changes in total phenolic contents during maturation are presented (Table 3). According to the results, the amounts of TP varied during maturity stages of *R. rugosa* hips with a statistically significant increase (*p* < 0.001) between maturity stages. Furthermore, the higher contents of TP were achieved at the ripe stage (red colour) with 21% of increase. TP accumulation in plants can be affected by genetic factors, environmental and cultural conditions as well as by various stresses [44]. These factors can explain the data differences reported within the scientific studies.

#### 3.5.2. Hydrophilic Antioxidant Capacity

During maturity, many biochemical, physiological and structural modifications occur, influencing the content of phytochemicals and thus affecting the antioxidant capacity. To the best of our knowledge, there are no previous studies to determine the variation of total antioxidant capacity in *R. rugosa* hips during ripening. Assays to measure total antioxidant capacity can be direct, which are based on the ability to inhibit the oxidation of a substance. The most common direct assays are H-TEAC and H-ORAC. As shown in Table 3, an increasing trend in the H-TEAC of *R. rugosa* hips was observed with significant differences (*p* < 0.001) between the different maturity stages. The highest activities were achieved at full maturity (red colour), the rate of increase was 67%. A possible explanation for these results is a correlation between bioactive compounds and antioxidant activities and this seems to be consistent with research done by [45]. Further analysis showed a positive correlation between TP and H-TEAC in *R. rugosa* hips during maturity (r = 0.9594). The observed increase in H-TEAC could be attributed to the contribution of predominant types of phenolic compounds at different ripening stages to overall antioxidant capacity. In the same vein, Müller et al. (2010) [16] suggested the major portion of antioxidant capacity was generated by polyphenolic compounds. On the other hand, the antioxidant capacity measured by the H-ORAC assay showed a significant variation (*p* < 0.001) as well. In fact, a decrease of H-ORAC values has been observed as the ripeness increased. These results may be explained by the fact that Folin-Ciocalteu and the radical scavenging method ABTS share the same reaction mechanism (electron transfer), whereas the H-ORAC method is based on hydrogen atom transfer reactions. The present findings seem to be consistent with Rodríguez et al. (2016) [46] who found the antioxidants capacity measured by H-ORAC significantly decreased as the palm fruit became ripe.

#### 3.5.3. Lipophilic Antioxidant Capacity

As mentioned above, rosehips are rich in lipid-soluble antioxidants such as carotenoids and tocopherols. The most abundant carotenoids were in the following order *(all-E)-*β-carotene, *(all-E)-* and *(Z)-*lycopene, followed by *(all-E)-* and *(Z)-*rubixanthin, *(all-E)-*zeaxanthin and *(all-E)-*β-cryptoxanthin. Among the different defence strategies, carotenoids are efficient deactivators of electronically excited sensitizer molecules that contribute to the generation of radicals and singlet oxygen. Moreover, carotenoids are involved in the quenching of reactive oxygen species, the molecular oxygen (^1^O_2_). For the physical scavenging, the efficacy of carotenoids is not only associated with the number of conjugated double bonds present in the molecule but also with the type of ring, functional groups on the rings, and so forth. [47]. Since vitamin E is a lipophilic molecule, its antioxidant functions are important for the protection of membrane lipids against peroxidation. α-Tocopherol is a chain breaking antioxidant capable of interrupting radical chain reactions produced by the lipid peroxyl radicals (LOO^•^). In addition, it directly scavenges superoxide radicals and singlet oxygen. As an antioxidant, the tocopherol reacts very quickly with peroxyl radicals to tocopheroxyl radicals due to its 6-hydroxychroman structure before it can abstract hydrogen from a target. The antioxidant capacity of the lipophilic extracts was determined using the L-TEAC method [17]. As a result, an increase in antioxidant capacity of lipophilic extracts (Table 3) was observed with statistical significance (*p* < 0.0001). The increase was progressed during maturity with +38% in orange colour and with +450% at full maturity (red colour). This finding confirms our previous results of accumulation of carotenoids during ripening.

## 4. Conclusions

This research extends our knowledge of rosehips, confirms previous findings and contributes additional evidence that suggests rosehips are a good source of carotenoids, vitamin E and vitamin C. Maturity stage affects the bioactive compounds as well as the antioxidant capacity in the fruit. Late harvesting is recommended if a high content of carotenoids is desire, while harvesting should be carried out earlier if higher vitamin E and vitamin C contents are desired.

## Figures and Tables

**Table 1:** Concentrations of carotenoids (mg/100 g) in saponified extracts of *R. rugosa* at different ripening degrees.

Carotenoid \ Sample	Green	Orange	Red
*(all-E)-*β-carotene	1.8 ± 0.1 ^a^	7.0 ± 0.4 ^b^	7.3 ± 0.2 ^b^
*(9Z)-*β-carotene	0.01± 0.0 ^a^	n.d.	0.1 ± 0.01 ^b^
*(13Z)-*β-carotene	n.d.	n.d.	0.3 ± 0.3
*(15Z)-*β-carotene	n.d.	n.d.	0.1 ± 0.01
*(all-E)-*α-cryptoxanthin	0.2 ± 0.01	0.2 ± 0.02	0.2 ± 0.01
(*all-E)-*β-cryptoxanthin	0.2 ± 0.02 ^a^	0.4 ± 0.04 ^b^	1.0 ± 0.03 ^c^
*(all-E)-*lutein	0.5 ± 0.03	0.5 ± 0.0	0.5 ± 0.01
*(all-E)-*lycopene	1.6 ± 0.2 ^a^	2.2 ± 0.4 ^b^	6.0 ± 0.6 ^c^
*(5Z)-*lycopene	0.4 ± 0.06 ^a^	0.6 ± 0.08 ^b^	1.4 ± 0.1 ^c^
*(9Z)-*lycopene	1.2 ± 0.1 ^b^	1.4 ± 0.2 ^b^	0.5 ± 0.05 ^a^
*(13Z)-*lycopene	4.2 ± 0.6 ^a^	6.0 ± 0.9 ^b^	6.5 ± 0.4 ^b^
*(all-E)-*rubixanthin	0.9 ± 0.1 ^a^	2.1 ± 0.2 ^b^	3.0 ± 0.1 ^c^
*(5′Z)-*rubixanthin	1.8 ± 0.04 ^a^	n.d.	3.6 ± 0.3 ^b^
*(all-E)-*violaxanthin	1.0 ± 0.3 ^a^	1.2 ± 0.4 ^b^	0.9 ± 0.4 ^a^
*(all-E)-*zeaxanthin	0.5 ± 0.1 ^a^	2.0 ± 0.1 ^b^	1.7 ± 0.0 ^b^
phytoene	0.8 ± 0.1 ^c^	0.6 ± 0.03 ^b^	0.4 ± 0.1 ^a^
phytofluene	n.d.	n.d.	n.d.

Data are expressed as mean ± SD (*n* = 3). One-way ANOVA with Student-Newman-Keuls post-hoc test, different letters a/b/c in the same line indicate significant differences (*p* < 0.05), n.d. not detected.

**Table 2:** Tocopherol and tocotrienol concentrations (µmol/100 g) in rosehips of *R. rugosa* at different ripening degrees.

Parameter \ Sample		Green	Orange	Red
α-Tocopherol	µmol/100 g	15 ± 3 ^a^	17 ± 2 ^b^	14 ± 1 ^a^
ɣ-Tocopherol		n.d.	n.d.	n.d.
∑ Tocotrienol		n.d.	n.d.	n.d.
∑ Vitamin E		15 ± 3 ^a^	17 ± 2 ^b^	14 ± 1 ^a^

Data are expressed as mean ± SD (n = 3). One-way ANOVA with Student-Newman-Keuls post-hoc test, different letters a/b within raw indicate significant differences (*p* ˂ 0.05). n.d. = not detected.

**Table 3:** Antioxidant capacity, total phenolic contents and ascorbic acid contents in *R. rugosa* hips at different ripening degrees.

Parameter \ Sample	Green	Orange	Red
Ascorbic acid (mg/100 g)	955 ± 71 ^b^	1090 ± 51 ^c^	798 ± 37 ^a^
Total phenolics (mg GAE/100 g)	1097 ± 123 ^a^	1009 ± 124 ^a^	1327 ± 43 ^b^
H-TEAC (mmol TE/100 g)	9.0 ± 1.0 ^a^	9.1 ± 1.0 ^a^	15 ± 1.0 ^b^
H-ORAC (mmol TE/100 g)	32 ± 4 ^b^	30 ± 4 ^b^	23 ± 0.3 ^a^
L-TEAC (mmol α-TE/100 g)	0.8 ± 0.2 ^a^	1.1 ± 0.1 ^a^	4.4 ± 0.3 ^b^

Data are expressed as mean ± SD (n = 3). One-way ANOVA with Student-Newman-Keuls post-hoc test different letters a/b within row indicate significant differences between samples (*p* ˂ 0.05). H: hydrophilic antioxidants, L: lipophilic antioxidants. GAE: gallic acid equivalents, TEAC: Trolox equivalent antioxidant capacity, ORAC: oxygen radical absorbance capacity, TE: Trolox equivalents, α-TE: α-tocopherol equivalents.
